# Ascribed Meaning of Disease Control: Perspectives of Patients With Type 2 Diabetes

**DOI:** 10.1177/2374373517745915

**Published:** 2017-12-14

**Authors:** Laura M Girling, Sarah E Chard, J Kevin Eckert

**Affiliations:** 1 Department of Sociology, Anthropology, and Health Administration and Policy, Center for Aging Studies, University of Maryland, Baltimore County, MD, USA

**Keywords:** diabetes, qualitative methods, health beliefs, control

## Abstract

**Background:**

Contemporary treatment of type 2 diabetes mellitus (hereafter diabetes) heavily emphasizes “diabetes control,” largely defined by measurable blood glucose parameters. Little is known about how people living with the condition themselves define diabetes control within the lived experience of their disease.

**Methods:**

As part of a qualitative study investigating the subjective construction of diabetes, 83 in-depth interviews were conducted with African American and non-Hispanic white older adults. Using content analysis, 4237 pages of narrative data were analyzed to explore how informants conceptualized diabetes control.

**Findings:**

Four themes emerged from the data, describing varied understandings of diabetes control: (a) blood sugar regulation, (b) practicable treatment adherence, (c) bodily experience, and (d) degree of pharmaceutical need. Findings demonstrate that among persons with diabetes, the term diabetes control is multifaceted.

**Conclusion:**

While clinical guidelines have established target blood glucose parameters as the standard indicator of diabetes control, persons with diabetes conveyed varied and diverse meanings situated within personal experiences. To foster empathetic and collaborative care, health-care providers tending to this population may consider integrating the emergent themes into communicative and treatment approaches.

## Introduction

Type 2 diabetes mellitus (hereafter diabetes), one of the most prevalent noncommunicable diseases, affects nearly 30 million Americans and ranks as the seventh leading cause of death in the United States (1). Anticipated to become an increasing public health concern, projections indicate as many as 1 in 3 Americans will have diabetes by 2050 (2). As diabetes becomes a mounting threat to the health and well-being of the population, there is a growing need to explore how individuals diagnosed with this illness understand their disease and terminology commonly associated with its description and treatment.

“Diabetes control” is a high priority and commonly used term within medical and public health communities, often noted as a cornerstone of diabetes care (3–7). Largely defined as an objective or measurable metabolic state, diabetes control is commonly gauged by achieving target blood glucose levels (eg, hemoglobin A_1c_ [HbA_1c_] < 7%) (8–10). Although the medical literature provides some standardization for this concept, parameters may vary depending on the type of test or cutoff scores utilized (10). Regardless of some variability, diabetes control is principally defined on the basis of metabolic indicators and is almost exclusively expressed numerically, emphasizing the pathophysiology of the disease (11–13). Although elected lifestyle behaviors offer a viable pathway, achievement of target glucose levels is complex, and in actual practice, quite difficult, with many patients failing to attain recommended parameters (10,14,15).

Although major clinical guidelines have established target glucose levels as the clear indicator of diabetes control, this concept may have little relevance to those afflicted with the disease as patients differ widely in their health beliefs and understanding of medical discourse. Research has shown that identical diabetes care terms can be used and interpreted differently by providers and patients (16–22). Aufseesser et al’s (21) evaluation of 8 medical terms related to diabetic retinopathy (eg, hemorrhage, microaneurysm) showed great inconsistency between patient and provider interpretations, indicating clinicians operate under disparate semantic assumptions than do those afflicted with diabetes. Likewise, using the Diabetes Semantic Differential Scales, Fitzgerald et al (20) found moderate differences in patient and provider perceptions on 5 of 18 diabetes care concepts (low blood sugar, diabetes complications, your emotions about diabetes, help with diabetes from family, and paying for diabetes). While providers utilized precise medical meanings, the authors suggest patients situate meaning within personal sociocultural worlds that often diverge from standard medical interpretations. Similar results have been found in other studies (19,22).

Despite a growing body of evidence proposing lay interpretations of common diabetes terms differ from clinical definitions, only a sparse body of literature has focused on arguably the most central concept within diabetes management, “diabetes control” (11–13,23–26). Existing qualitative research suggests patients’ interpretations of diabetes control extend standard medical definitions to include practical and experiential designations. A noteworthy study (11) conducted in the late 1990s with self-identified Mexican and Mexican Americans found patients’ central criteria for evaluating diabetes control were based on how healthy they felt (eg, having energy, absence of dizziness) and/or how well they were able to maintain normal activities. A more recent mixed methods study (25) that focused on impediments to glycemic control found that while a majority of patients reported knowing their HbA_1c_ value, *not one* patient voluntarily cited HbA_1c_ as the primary measure of diabetes control. Despite important insights presented in prior research, our review indicates much of what is known about personal meanings of diabetes control is dated (<10 years), outside of US contexts, or focuses on small samples with limited narrative reflecting patients’ voices and their nuanced interpretations. The primary purpose of this study is to expand upon the limited, qualitative information on how persons with diabetes define diabetic control within the lived experience of their disease.

## Methodology

### Sampling and Participants

**Figure 1.**
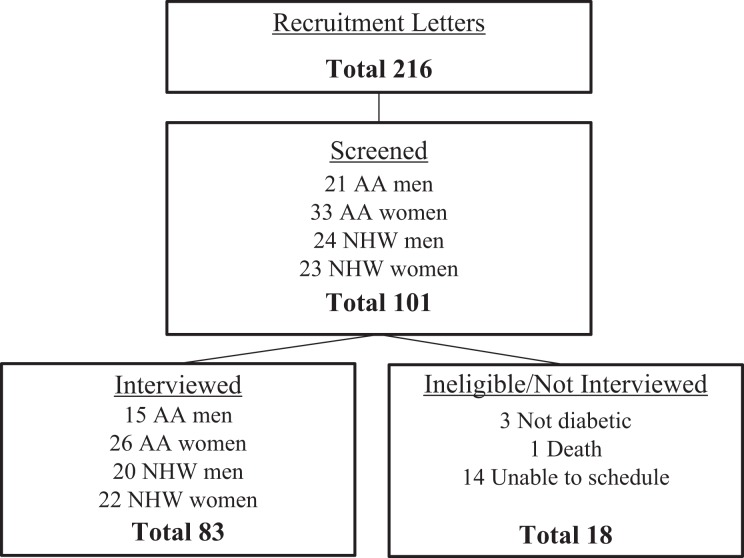
Recruitment flowchart.

Data for this analysis were drawn from the *Subjective Experience of Diabetes among Urban Older Adults* (SED) study (27), a qualitative project exploring subjective understandings of diabetes among male and female African American (AA) and non-Hispanic whites (NHW). The community-dwelling sample was recruited through the *Healthy Aging in Neighborhoods of Diversity across the Life Span* (HANDLS) study (28). Eligibility criteria were a clinical diabetes diagnosis and age ≥50. In waves, interest letters were mailed informing eligible participants about the opportunity to enroll in SED. Of the 216 letters mailed, 101 individuals contacted the study’s staff. Of the 101 interested callers, 83 were interviewed. As depicted in Figure 1, 18 interested individuals were not interviewed for various reasons. During the brief phone screen, 3 indicated they did not have diabetes, 1 person died prior to scheduling an interview, and 14 were unable to schedule because of either health reasons (eg, recovering from surgery) or loss of interest. The final sample included a total of 83 informants. Demographic information is detailed in Table 1.

**Table 1.** Informant Demographic Characteristics.^a^

Characteristic	AA, n = 41		NHW, n = 42	
	Mean (SD)	Frequency (%)	Mean (SD)	Frequency (%)
Age	61.5 (5.6)		60.9 (6.3)	
Completed education, years	12.3 (1.9)		11.3 (2.6)	
Monthly income, US$	1804 (1451)		2563 (2179)	
Gender				
Male		15 (36.6)		20 (47.6)
Female		26 (63.4)		22 (52.4)
Diabetes duration, years^b^				
≤1		0 (0)		1 (2.4)
1-4		3 (7.7)		2 (4.8)
5-9		12 (30.8)		9 (21.4)
≥10		24 (61.5)		29 (69.0)

Abbreviations: AA, African American; NHW, non-Hispanic whites; SD, standard deviation.

^a^N = 83.

^b^n = 80, data unavailable for 3 informants.

### Data Collection

Following the informed consent process, experienced ethnographers conducted a single tape-recorded, in-depth, semi-structured interview with each informant. Ethnographer and informant were matched by race, when possible, as a technique to offer informants increased comfort and opportunity for disclosure (29,30). The semi-structured interview utilized a modified version of the McGill Illness Narrative Interview (31), a theoretically driven qualitative protocol specifically designed to elicit illness narratives. Interviews averaged 100 minutes and were conducted in participant’s homes, unless an alternate location was requested. After each interview, ethnographers recorded field notes to expand audio-recorded data with enriched descriptions of nonverbal behaviors, physical characteristics, and reflective summaries (32). Interviews and field notes were professionally transcribed and de-identified. Informants received a US$50 cash honorarium for their participation. All names and locations were changed to protect the identity of informants under a protocol approved by the institutional review board at the University of Maryland, Baltimore County.

## Analysis

### Coding

To systematically explore over 4000 pages of narrative, data were formatted into ATLAS.ti (33), an organizational and analytical program that expedites evaluation of complex phenomenon in unstructured data. Analysis initiated with the development of an inductively derived codebook (27). First, the entire research team open-coded a selection of transcripts (n = 8; 2 AA men, 2 AA women, 2 NHW men, 2 NHW women) to identify a preliminary set of codes. This intensely iterative process involved an inventory or a raw sort on a gross level of categorization providing an opportunity for ongoing discovery and refinement (34). Transcripts were cross referenced with discussion from team meetings and field observations in order to distill a draft schema of 49 codes. After development of the initial coding schema, the study team tested the codes by applying them to an additional subset of transcripts (n = 20). Examination of the effectiveness and applicability of the developed schema led to further refinement which resulted in a final codebook of 40 mutually agreed-upon codes. Coding proceeded with rotating teams of 2: team members independently coded the same transcript line by line in ATLAS.ti, followed by in-person paired review to reconcile discrepant coding. Unresolvable differences were brought to team meetings for a consensus resolution. The systematic process of dual coding among rotating pairs and team-based resolution is designed to reduce bias and provide an important validity check (35).

### Thematic Development

For this analysis, all data coded “Health Beliefs” (internalized health beliefs, biomedical, alternative, subjective, folk, complementary, and alternative medicine) were retrieved to explore patterns in how diabetes control was discussed by informants. This code captured spontaneous reflection about disease control as well as direct questioning on the topic: (a) “What does control mean to you?” and (b) “Do you feel you have control over your diabetes?” The retrieved textual data were read in its entirety by the lead author, with the coauthors assigned to equally divided narrative segments. The data underwent an iterative examination where the authors read, reflected upon, reread, and reconsidered the narrative identifying recurrent patterns in conversational topics and meanings. To enhance the quality of the findings, patterns were constantly checked against the text, the context, prior elucidations, and across author’s interpretations. As a validity check, the authors actively sought confirming and disconfirming evidence throughout this process (35). Themes that recurred regularly confirmed original observation (36,37). Recurrent patterns in conversational topics and meanings, as presented in informant’s own view in direct statements, were organized to formulate focal themes. This analytic process was repeated through several cycles until a final thematic consensus was reached between authors, resulting in 4 central themes.

## Findings

Diabetes control was described in highly personal and individualized narrative, which can be clustered into 4 broad thematic categories: blood sugar regulation, practicable treatment adherence, bodily experience, and degree of pharmaceutical need. The following quotes were selected as exemplars, portraying the nuanced statements relating to differing meanings of control.

### Blood Sugar Regulation

The first ascribed meaning of diabetes control, blood sugar regulation, encompassed a medicalized understanding of the concept, harmonious with clinical definitions. This meaning was primarily voiced using numeric designations. An example can be seen within Arthur’s interview:

Interviewer: So what does the word control mean to you?

Arthur: I guess you’re within the limits of where they want you to be with numbers. It’s pretty much a numbers game, diabetes I guess, because they want you to be at a certain number all the time…I’m saying it was 272 when I went to HANDLS, it was 454 mg 6 months ago. I didn’t feel any different, felt the same then as I felt when I went to HANDLS so even though the number was outrageous and they were flipping out, I didn’t feel any different.

Characterized by measurable glucose parameters, this conceptualization demonstrates a shared meaning between lay and clinically trained populations. Although some confidently echoed their doctor’s numeric definition of diabetic control, such definitions also lead to puzzlement regarding the physiological significance. As seen in Arthur’s narrative, despite experiencing alarmingly high glucose levels, he described feeling physically well. Much of the language expressed within such depictions suggests a distinction between one’s subjectively understood health and the physiology of the diabetic body, as the following example further illustrates:

I felt good all that day, and when it was time to go to bed and I went to check my sugar and the meter read 500…you know what I said? I said wow, I said that is incredible. I wasn’t afraid, I was like that is incredible. I said talk about a silent killer.

Although informants were largely aware of the clinical definition of diabetes control, often reiterating parameters discussed in clinical encounters, their subjective interpretations point to the biological complexity of diabetes, specifically the incongruence between numerically evaluated glycemic control and the lived experience of the body. Such incongruence reinforced the inclination to define control through glucose parameters as such indicators were perceived as both reliable and objective.

### Practicable Treatment Adherence

Practicable treatment adherence included descriptions of control in terms of complying with medicinal, dietary, and/or physical activity recommendations by various providers, including dieticians, nutritionists, endocrinologists, and primary care doctors. When directly asked what diabetes control meant to her, Celeste explained:

What control means? That stay on your insulin, stay on your medicine, take your medicine when you’re supposed to take it and check your sugar and stuff properly and stay on your insulin. They say you take 46 units, TAKE 46 units. They say you take 30 units, TAKE 30 units. If they say you take a certain amount of units, take it, that’s all you got to do is stay on your medicine and obey the doctor and visit your doctor every time they give you an appointment, do not ignore your doctor, that’s the worst thing to do is ignore your doctor [endocrinologist].

This depiction of diabetes control focuses heavily on treatment adherence, specifically following endocrinologist’s medicinal intake, glucose monitoring, and appointment maintenance recommendations. To participants such as Celeste, the clinical definition of diabetes control was acknowledged, but interpretations went beyond achievement of target glucose parameters to include meanings of adherence. It is important to note this theme incorporates acknowledgment that people act as owners of their own bodies. Diabetes control was not discussed as blindly following health-care provider’s instructions but was framed around the notion that providers are trained to offer guidance that can be integrated into one’s diabetes care routine. Rather than complete adherence, informants discussed a proclivity to realistically fit recommendations into a real-life context, making them more practically manageable within daily routines. For instance, informants recalled altering the timing or amount of insulin injections to accommodate social or work events. Many acknowledged that completely adhering to strict dietary and pharmaceutical recommendations was both unrealistic and unfeasible, given that many factors compete for the time and attention arduous diabetes treatment regimens require.

### Degree of Pharmaceutical Need

Diabetes control was also conceptualized across a spectrum of pharmaceutical need. For some, diabetes control meant (a) the absence of pharmaceutical treatment or (b) minimal treatment as perceived by either (i) dosage or (ii) modality.

Among those who viewed diabetes control as the *absence* of pharmaceutical treatment, depictions included simple yet poignant statements such as “not needing medicine.” When discussing her understanding of diabetic control, Beverly notes: “…eliminating the medication. But my day, I’m looking forward to the day that I don’t have to take the medication anymore.” Beverly conceptualizes diabetes control as a point in her disease course when she no longer requires pharmaceutical treatment of any kind, highlighting a common desire to live “medication free.” However, reaching such control was deemed by informants, with few exceptions, to be an idealistic goal.

Similar to the absence of medicinal therapy, diabetes control was also depicted in terms of minimal pharmaceutical need—specifically in relation to *dosage* or *modality*. In terms of minimal *dosage*, when asked why she felt her friend’s diabetes was controlled, Evelyn stated:

To me I felt hers was well under control because the dosage pill that she was taking was like a 30 mg pill, you know, and I said well, you doing good I said because you’re on nothing like what I was taking for mine…

Evelyn depicts diabetes control as requiring only low dosages of oral diabetes medication. While the exact units varied by person, this theme was not only apparent among oral agent user but also visible among those utilizing injection therapies; lower units perceived as a reflection of disease control.

Paralleling dosage, minimal treatment as expressed by therapeutic *modality* also emerged as a meaning of control. For many, diabetes control incorporated the prescription of oral medication only, whereas necessity of insulin injections represented a lack of disease control. When asked to clarify her understanding, Mary Beth specified that when insulin injections are integrated into a treatment regime, one has progressed far into their disease where diabetes control cannot be obtained:

…if your disease progresses, you progress from your oral [metformin] to your insulin, you know, but I’m saying, you know, that possibly like that’s one thing. You were saying is there a difference. It could be that, you know, the illusion that you’re not as bad off because you’re not on insulin.

Within this conceptualization, the meaning of diabetes control goes beyond metabolic indicators to incorporate descriptions of treatment gravity. Regardless of whether glucose levels were within or outside target parameters, dependence on oral medication (most often metformin) was perceived as a minimal mode of treatment and a reflection of diabetes control, whereas prescription of insulin was viewed as a more intense form of treatment that signified a lack of disease control. For instance, despite experiencing high or low glucose values, some informants deemed their diabetes to be controlled because insulin injections were not a component of their treatment regime.

### Bodily Experience

The final emergent meaning included diabetes control as defined by bodily experiences. More specifically, some informants extended their understanding of diabetic control beyond target glucose levels to include feeling physically well in relation to diabetes complication and/or symptoms. When asked what diabetes control means, Alberta stated:

Control means not being sick, not experiencing the painful symptoms, nausea, fatigue, neuropathy, disease control is about feeling normal despite the diagnosis [diabetes].

Reinforced by means of nonverbal communication, Alberta connects her bodily experience and diabetes control: “Cause she see that, you know, it’s under control [pointing to her whole body].” This collective interpretation acknowledges a physical meaning of control, irrespective of numeric indicators, giving priority to the physical experience of diabetes. Of note, the body’s wellness was perceived as the central criterion for evaluating diabetic control, indicating control was not defined as a separate imperceptible attribute, as many times glucose values can be.

## Discussion

“Diabetes control” is a cornerstone of diabetes care and is viewed as the ultimate clinical outcome within diabetes management practices. In clinical contexts, diabetic control generally refers to glycemic control, defined by objective and measurable metabolic conditions. However, prior research demonstrates clinically defined diabetes care concepts may have little relevance to those afflicted with the disease (20–22).

Unlike Elliott et al’s (25) study where *not one* participant volunteered HbA_1c_ as the primary indicator of diabetes control, informants in our sample widely described control consistent with clinical definitions (eg, glucose parameters). Although research demonstrates diabetes care concepts can be interpreted differently by patient and provider populations (19–22), the tendency of our sample’s descriptions to align with clinical definitions (glycemic control) is perhaps unsurprising as diabetes control is not a peripheral concept but a term central to contemporary diabetes management practices (13). Despite attunement to clinical definitions of diabetes control, such depictions were accompanied by confusion about the relationship between numerically evaluated diabetes control and the lived experience of the body. Informants reported feeling “great” even when blood glucose levels were alarmingly high (eg, >500 mg/dL). Paralleling other research (13,38), such puzzlement led many informants to view objective measures, such as glucose parameters, as more reliable determinants of diabetes control, as compared to subjective feelings. Montez and Karner (13) as well as Frank (38) posit that when metabolic indicators take precedent over subjective experiences, it results in distrust of the diabetic body and a body-self separation.

Contrasting this body-self separation, for some informants, diabetes control was recognized as a physically experienced phenomenon, a merging of the body and self. This lay understanding suggests that for some individuals, bodily experiences are central to their interpretation of disease control, whereas glucose parameters function as a more peripheral component. Similarly, Hunt et al (11) and Elliott et al (25) found many in their respective samples evaluated physical experiences (eg, having energy, feeling well) as central conditions for assessing diabetes control. While Elliott suggests such conceptualization reflects a “lack of understanding,” Hunt advocates that experiential interpretations reflect personal models of illness that seek to connect body and outcome.

Unobserved in prior qualitative evaluations, many informants identified pharmaceutical need or treatment adherence as central to their understanding of diabetes control. Interestingly, depictions of diabetes control in relation to pharmaceutical need (dosage or modality) resemble the “treat-to-failure” philosophy (39,40), a treatment paradigm of sequential monotherapy. In this philosophy, patients are given the least intense therapy first, followed by medications of increasing intensity as the disease worsens, with insulin considered the last resort. It is conceivable that experiencing this stepwise approach to prescribing medication may have influenced how patients interpreted diabetes control. However, based on interview data alone, it is unknown whether informant’s health-care providers utilized this method in their clinical practice.

Also unique to the qualitative exploration in this area, many informants described the concept of diabetes control in relation to adhering to clinical recommendations. Although superficially resembling a paternalistic model of health care (41), where patients yield to professional’s choice in treatment, this theme went beyond thoughtlessly following recommendations to include a proclivity to realistically fit care plans into a real-life context. While there is some controversy surrounding the extent patients should modify treatment approaches (42), the notion that some persons with diabetes define diabetic control by strategically and thoughtfully adapting care plans to achieve balance between larger life contexts and illness demands highlights the unique ways this population contend with such a complex disease.

### Limitations

This analysis only reports findings from interviews with AA and NHW men and women who were recruited from 1 general geographic area. Although the sample was properly composed to meaningfully explore subjective experiences among 2 racial groups in a mid-Atlantic region of the United States, the generalizability of the findings across diverse populations is limited. Because the study focused on qualitative objectives, clinical measures of diabetes control were not assessed and were not obtainable through the recruitment study. Consequently, we were unable to explore individual perceptions or experiences in relation to clinical indicators (eg, HbA_1c_). Additional studies are needed to understand perceptions of diabetes control with respect to clinical measures as well as among other minority groups who are disproportionately affected. Future research could extend these findings by exploring diabetes control among persons of Hispanic and Latino origin as well as those with varying degrees of clinically evaluated glycemic control.

## Conclusion

Despite semantic similarities, there are many important differences in how persons living with diabetes subjectively interpret the meaning of diabetic control, of which health-care providers should be cognizant. Because divergent beliefs have been shown to negatively affect health care (43,44), attunement to nuanced interpretations could lead to collaborative alliances between patient and providers as well as congruent objectives and improved health outcomes. For instance, within the clinical encounter, if a provider uses the term diabetes control to denote glycemic control, they may consider whether the patient comprehends the intended connotation and to inquire about their personal interpretation. If a patient interprets diabetes control in relation to the dosage or modality of medication, the health-care provider could encourage the patient to pursue the clinical definition (target glucose levels) while simultaneously strategizing to attain the patient’s personal model of diabetes control (desired degree of medicinal dependence).
